# Teaching during COVID-19: reflections of early-career science teachers

**DOI:** 10.1186/s43031-022-00057-y

**Published:** 2022-04-15

**Authors:** Jeanna R. Wieselmann, Elizabeth A. Crotty

**Affiliations:** 1 Southern Methodist University, Dallas, TX 750455 USA; 2 University of Wisconsin – Eau Claire, Eau Claire, WI USA

**Keywords:** Teaching during COVID-19, Distributed leadership, Technology leader practices, Emergency remote teaching, Teach for America, Early-career teachers

## Abstract

The unique circumstances of the COVID-19 pandemic required that instruction be shifted online through asynchronous, synchronous, or hybrid models of instruction. This created a need for many K-12 teachers to dramatically rethink how teaching and learning occurred in their classrooms. In this study, we investigate the experiences of early-career science teachers who were in their first year of teaching when the pandemic struck. Using a comparative case study and an analytical framework focused on technology-related leader practices, we explore the unique opportunities for technology-based leadership that emerged for early-career teachers during the pandemic. We posit that the circumstances of the COVID-19 pandemic presented novel opportunities for early-career teachers to assume leadership roles that were embedded within the classroom teaching experience, which created unique opportunities for innovation and leadership in teaching.

Spring of 2020 brought about unprecedented challenges for people around the world, including teachers who faced extended school closures due to the COVID-19 pandemic. In a matter of days, many teachers transitioned from in-person teaching to fully remote instruction, often lacking clear guidance and support in doing so. Decades of research in online and distance learning have revealed the complexities of online instruction (Means et al., 2014). Although studies have demonstrated positive outcomes associated with online and distance learning (e.g., Chang, 2016), a number of personal and contextual factors influence learning outcomes (Panigrahi et al., 2018). Further, quality online courses require months of preparation (Hodges et al., 2020). This was not possible given the COVID-19 circumstances; rather, teachers and students faced a transition to emergency remote teaching (ERT). ERT represents a rapid shift in instructional delivery mode that is temporary in nature, providing short-term access to instruction that would not otherwise be available (Hodges et al., 2020).

Such rapid and significant shifts in teaching are unprecedented, and technology integration requires intentional planning (Cox, 2013). Even in less extreme circumstances, the importance of leadership in supporting teachers’ use of technology is clear. Without adequate training and support, teachers tend to utilize technology to present content, rather than to engage students in deeper, student-centered learning activities (e.g., Lim & Chai, 2008; Ward & Parr, 2010); this is particularly true among beginning teachers (Tondeur et al., 2017). To move beyond simply using technology as a presentation tool and integrate technology more deeply into instructional practices, teachers and leaders need to work together to identify opportunities for using technology to meet instructional goals (Dexter & Richardson, 2020). Unfortunately, given the rapid nature of the shift to ERT in 2020, this intentional planning and professional learning was not possible.

This study explores the experiences of a particular group of teachers: Teach for America (TFA) corps members who were in their first year of teaching science to students in grades 5–12 in spring of 2020 and finished their two-year TFA commitment amidst the ongoing challenges of the 2020–2021 school year. The following research questions are addressed:

1. How do first-year science teachers describe their experiences shifting to emergency remote teaching during COVID-19?
2. How do early-career teachers experience and enact technology-related leader practices during emergency remote teaching associated with COVID-19?

## Literature review

### Teacher effectiveness and retention

Science teachers today are tasked with providing students hands-on learning experiences to promote their development of science content knowledge, science and engineering practices, and crosscutting concepts that span disciplines (National Research Council, 2012; NGSS Lead States, 2013). This is a complex task that requires strong pedagogical content knowledge (van Driel et al., 1998), and concerns about teachers’ science backgrounds persist. For example, as many as ^1/3^ of high school environmental science teachers have not had any college coursework in their field (Banilower et al., 2018). Further, the most qualified teachers are not distributed equitably across school contexts, with those classes with high-achieving students and higher socioeconomic status more likely to have teachers with strong content backgrounds (Banilower et al., 2018). This is problematic because teacher preparation is related to teacher effectiveness (Darling-Hammond et al., 2005).

Highly prepared teachers are less likely to leave the teaching profession (Boyd et al., 2011), and teachers also tend to become more effective over time as they gain teaching experience (Harris & Sass, 2011; Papay & Kraft, 2015), making teacher retention an important factor related to instructional quality. A variety of studies have found that alternative certification programs that provide a range of different preparation experiences for teachers, including TFA, have higher rates of teacher attrition than traditional teacher preparation programs (e.g., Goldhaber & Cowan, 2014; Redding & Smith, 2016), with more than 80% of TFA teachers leaving the profession within 3 years (Heilig & Jez, 2010). TFA corps members who do not have a teaching certification are also less effective than certified teachers (e.g., Darling-Hammond et al., 2005; Heilig & Jez, 2010). Thus, TFA corps members face the typical challenges of early-career teachers, as well as additional challenges related to their abbreviated preparation process.

With teacher turnover concerns both with TFA teachers and in general, providing early-career teachers authentic opportunities for leadership can be one means of supporting their persistence in the field of teaching. York-Barr and Duke (2004) noted that teacher leadership often provided new opportunities for teachers to feel reinvigorated in their work and helped with teacher retention; however, this model of leadership often required teachers to leave their classroom positions to engage in formal leadership positions. In this paper, we posit that the circumstances of the COVID-19 pandemic presented novel opportunities for early-career teachers to assume leadership roles that were embedded within the classroom teaching experience, which created unique opportunities for innovation and leadership in teaching.

### Professional phase and technology integration

Beginning teachers often still have a lot to learn about classroom teaching, but also bring technological expertise for online instruction that many veteran teachers may not have. Day and Gu (2007) found that teacher identity was informed by two primary things: 1) the teacher’s sense of positive professional identity and 2) their professional life phase. Day and Gu (2007) identified that the professional life phase of years 0–3 in teaching could be categorized in two sub-groups, either developing a sense of efficacy or reducing a sense of efficacy. Activities focused on professional learning in relation to classroom knowledge, specifically from colleagues in the school, seemed to have the largest influence on beginning teachers; thus, learning opportunities that build a sense of professional identity and classroom competence are critical for teachers in their first 3 years (Day & Gu, 2007).

Beginning teachers who are part of Gen Y and had exposure to technology as students seem to be well equipped for technology integration in teaching compared to their more experienced colleagues (Gao et al., 2010; Wang et al., 2014). However, previous studies have produced different findings related to the nature of technology integration among early-career teachers. For example, Goos (2005) found that beginning teachers were better able to integrate technology in diverse and flexible ways for student learning and were more willing to do so. In contrast, Russell et al. (2003) found that despite higher levels of comfort with technology, new teachers were more likely to use it for planning purposes, while more experienced teachers reported using technology during student instruction. Similarly, Lei (2009) found that teachers who were digital natives lacked proficiency with more advanced technologies. Tondeur et al. (2017) found that technology-related experiences in teacher preparation programs, including technology-specific coursework, modeling of technology use during internships and field experiences, and feedback from mentors on technology use, influenced the degree to which early-career teachers were prepared to integrate technology. Despite the lack of certainty about the extent to which early-career teachers utilize technology in their instruction, those who have specialized knowledge and skills for teaching with technology may also have unique opportunities for teacher leadership early in their teaching careers (Gao et al., 2010; Gao et al., 2011).

### Leadership practices for technology integration

Teachers’ technological skills and knowledge shape their ability to lead instructional practices with technology. Öqvist and Högström (2018) found that when teachers had limited technology expertise, their technology leadership with children was hindered, and they were less willing to encourage exploration and open inquiry with technologies in school. Teachers having a positive mindset about technology and a strong sense of self-efficacy are necessary for driving technological leadership in schools (Pan & Franklin, 2011). In Dexter and Richardson (2020) review of the research literature framing technology integration leadership, several key themes were relevant to leadership practices for technology integration. One of the dominant themes from their review was the idea that teachers and leaders need to have opportunities to learn alongside each other and apply technology to their instructional goals. Another goal was described in developing a collaborative leadership space in which cognition was distributed across individuals, with teachers with more technological expertise mentoring teachers who were less skilled (Dexter & Richardson, 2020.

## Theoretical and analytical framework

Teacher leadership has been defined in many ways within the research literature and within schools, from formal leadership roles like department and grade-level leads to more distributed leadership approaches, where the daily work that teachers do is recognized as practical, yet critical, informal leadership that can have significant influence on a school’s culture and climate (Silva et al., 2000). While there are many different definitions of teacher leadership, we are adopting the definition posed by Spillane et al. (2003) of school leadership as “the identification, acquisition, allocation, coordination, and use of the social, material, and cultural resources necessary to establish the conditions for the possibility of innovation in teaching and learning” (p. 535). Under this definition of distributed leadership, the practices of a few select teachers have the potential to mobilize changes in teacher practices and create innovations in instruction.

Collaborative and distributed leadership models create opportunities for teachers to learn from each other’s practices. Lieberman and Friedrich (2010) emphasize that teacher leadership is most effective when leadership comes in the form of working “alongside” teachers in a collaborative effort and when instructional practices are made public for teachers to learn from one another. The leadership that emerged during the COVID-19 pandemic often resulted from teachers sharing their practices for online teaching and through ongoing collaboration (Gandolfi & Kratcoski, 2020). Berry et al. (2013) call for the blurring of the lines between teaching and leadership, where teachers lead and leaders teach in order to truly impact professional practice.

School leaders play a key role in supporting teachers and thereby improving student outcomes (e.g., Hallinger & Heck, 2010; Supovitz et al., 2010). Previous studies have found that school principals contribute nearly as much to student achievement as classroom teachers do (Grissom et al., 2021; Leithwood et al., 2020). Hitt and Tucker (2016) synthesized 56 empirical research studies and three frameworks (Leithwood, 2012; Murphy et al., 2006; Sebring et al., 2006) in an effort to identify leader practices that influence student achievement. From their review, five domains comprising a total of 28 key practices emerged, resulting in the unified model of effective leader practices (see Table 1). Notably, while expected of formal school leaders such as principals, these practices are often distributed across informal leaders as well (Hitt & Tucker, 2016). Thus, a range of individuals may enact these leader practices at the classroom, school, and community levels.

**Table 1:** Unified model of effective leader practices

Domain	Leader Practices (Hitt & Tucker, 2016)	Operationalization of Technology Leader Practices During COVID-19 (adapted from Dexter et al., 2016)
Establishing and conveying the vision	● Creating, articulating, and stewarding shared mission and vision ● Implementing vision by setting goals and performance expectations ● Modeling aspirational and ethical practices ● Communicating broadly the state of the vision ● Promoting use of data for continual improvement ● Tending to external accountability	● Identifying vision for online and hybrid instruction during COVID-19 ● Setting goals and performance expectations related to the use of technology ● Modeling use of technology ● Promoting the use of data to improve technology-based instruction
Facilitating a high-quality learning experience for students	● Maintaining safety and orderliness ● Personalizing the environment to reflect students’ backgrounds ● Developing and monitoring curricular program ● Developing and monitoring instructional program ● Developing and monitoring assessment program	● Utilizing technology to promote student engagement while learning online ● Maintaining safe and organized online learning settings ● Developing and monitoring use of technology
Building professional capacity	● Selecting for the right fit ● Providing individualized consideration ● Building trusting relationships ● Providing opportunities to learn for whole faculty, including leader(s) ● Supporting, buffering, and recognizing staff ● Engendering responsibility for promoting learning ● Creating communities of practice	● Providing technology-focused professional learning opportunities for faculty and staff ● Supporting and recognizing exemplary uses of technology
Creating a supportive organization for learning	● Acquiring and allocating resources strategically for mission and vision ● Considering context to maximize organizational functioning ● Building collaborative processes for decision making ● Sharing and distributing leadership ● Tending to and building on diversity ● Maintaining ambitious and high expectations and standards ● Strengthening and optimizing school culture	● Acquiring and allocating technology resources to teachers and students ● Attending to equity in technology access ● Distributing technology-related leadership among faculty and staff ● Tending to and building on teachers’ diversity of experiences with technology ● Maintaining high expectations for technology integration ● Supporting teachers in collaboration focused on technology
Connecting with external partners	● Building productive relationships with families and external partners in the community ● Engaging families and community in collaborative processes to strengthen student learning ● Anchoring schools in the community	● Utilizing technology to build relationships with families and the community ● Engaging families in collaborative processes to strengthen student learning via technology

The first domain, establishing and conveying the mission and vision, includes six practices, such as setting goals and performance expectations related to the vision, modeling the vision, and promoting the use of data for continuous improvement. Second, facilitating a high-quality learning experience for students contains five practices, including maintaining safety and orderliness, personalizing the environment to reflect students’ backgrounds, and developing and monitoring curriculum, instruction, and assessment. Third, building professional capacity contains seven practices, including hiring faculty and staff that are a good fit, building trusting relationships, providing professional learning opportunities, and creating communities of practice. Fourth, creating a supportive organization for learning consists of seven practices, including acquiring and allocating resources, tending to and building on diversity, sharing and distributing leadership, and maintaining high expectations. Finally, connecting with external partners features three practices, such as building productive relationships with families and the community and engaging families and community in collaborative processes to strengthen student learning.

Dexter et al. (2016) adapted Hitt and Tucker’s (2016) five domains of leadership to focus specifically on the integration of technology into instruction. For example, in facilitating a high-quality learning experience for students, Dexter et al. (2016) focused on the use of technology in these learning experiences. Building professional capacity includes specific attention to developing teachers’ capacity to integrate technology in their instruction. Creating a supportive organization includes attending to the needs associated with technology integration. Building on the work of Hitt and Tucker (2016) and Dexter et al. (2016), we have operationalized the domains and leader practices for the current study as shown in the right column of Table 1.

## Methods

A comparative case study (Yin, 2014) was used in this work to explore the phenomenon of being an early-career science teacher during COVID-19. A total of 13 teachers who were enrolled in a masters-level course focused on differentiated instruction within the context of science teaching participated in this study. The participants included eight women and five men currently teaching in the South-Central region of the United States. They ranged in age from 21 to 23 years. These individuals completed their bachelor’s degrees in the spring of 2019 at a range of public and private institutions across the United States. They participated in TFA training and coursework over the summer of 2019, then started as science teachers of record in fall of 2019.

Data collection and analysis proceeded in two phases. First, all 13 participants completed written reflections about their first-year teaching experiences within the context of COVID-19, which was part of an assigned end-of-semester paper completed in May of 2020. Participants were instructed to write about the following topics:

- Reflections on transitioning to online instruction because of COVID-19
- Challenges faced in online instruction
- Successes experienced in online instruction
- Lessons learned or things to transfer from online teaching to in-person teaching in the future
- Ideas or vision for how the broader education system might change as a result of the widespread use of online teaching during COVID-19

To analyze these written reflections, we utilized inductive coding (Saldaña, 2016) across two independent coders to identify key themes related to early-career teachers’ experiences teaching online during COVID-19. Following an initial cycle of open coding, the researchers developed a codebook and operationalized each code, including a definition and examples from the dataset. A second cycle of coding followed, during which we applied the defined codes to the teacher reflections. We met to discuss all coding discrepancies and came to consensus on each coded segment. This initial phase of data analysis allowed us to identify both similarities and differences in teacher experiences, key elements in comparative case study (Yin, 2014).

Based on the first phase of data collection and analysis, we purposefully selected two teachers for further study based on the unique technology-related leadership experiences they wrote about. These participants were selected based on their discussion of leadership opportunities related to technology, as well as their willingness to participate in additional interviews. We conducted semi-structured interviews with these teachers over the summer of 2021. We then transcribed the interviews and analyzed the data using deductive coding based on the domains and dimensions of the unified model of effective leader practices (see Table 1). Two researchers coded the transcripts independently, then met to address all differences in coding and reach consensus. We also applied this deductive coding process to the written reflections of these two participants. Throughout this process, constant comparative analysis strategies (Corbin & Strauss, 2015) were used to identify consistencies and differences across cases.

## Findings

### Phase 1: experiences of first-year teachers shifting to online instruction

Drawing upon the data collected from all 13 participants, three key themes were identified in the teachers’ written reflections. Discussed in the following sections, these themes include shifting approaches to teaching, beliefs about students, and unique opportunities that emerged because of the COVID-19 circumstances.

#### Approach to teaching

As they transitioned from in-person to ERT, first-year science teachers described growth in their teaching practices in a number of ways. With state standardized tests being canceled due to COVID-19, teachers felt free to deviate from a test-preparation focus and became more creative in their lesson planning. They described shifting from test-focused instruction to more student-centered instruction. One teacher reflected, “I was able to shift my focus from being test-focused to being able to focus more on my students’ needs and interests.” Another teacher shared, “I am using this time to try and give them more explorative opportunities, which I think is at the core of STEM learning.”

Teachers reported planning a range of innovative activities. For example, they developed hands-on investigations students were able to complete using common at-home materials. One teacher challenged students to develop a model of something they had studied over the course of the school year, then led “Show Off Friday” meetings in which students shared their models with the class. Another teacher created screen capture recordings of her illustrating and describing complex science concepts (for example, the water cycle), focusing on developing her students’ understanding of systems. Another participant designed a virtual escape room activity that required students to use everyday materials and their application of science concepts to “escape” a virtual room. These activities represented a marked departure from their standard in-person practice, which often emphasized lecture, note-taking, and practice test questions. Real-world relevance became a central factor in the lessons teachers prepared, with teachers reporting connecting their instruction with the students’ home and community environments, as well as the COVID-19 pandemic, which offered many opportunities to learn about science concepts and the nature of science.

Notably, these innovations were not successful in every instance. However, the teachers described recognizing and valuing the experience of trying something and failing, then using that failure to inform their subsequent lesson plans. As teachers experimented with instructional approaches that did not rely on lectures and worksheets, they reported that many students benefited, as illustrated by some increasing their participation and engagement over what was typical of in-person instruction. In comparing her ERT student participation rates to the participation rates her colleagues encountered, one teacher described, “I have had a tremendously high engagement rate compared to my team and school, at around 90% of students participating in eLearning.”

In addition to shifting their instructional practices, participants also reported adjusting their approach to communicating with students’ families. One teacher described, “I have been able to talk to so many of my students’ families during this time and build relationships.” Another reflected that although these increased communications were initially mandatory, she found them to be incredibly valuable. Many teachers recognized that these connections built a strong foundation for a collaborative approach to student learning, and they planned to bring them into the next academic year.

#### Beliefs about students

Some teachers found their beliefs about student motivation shifted as a result of online teaching. For example, one of the districts passed a “no zero” policy that would prevent students from failing any courses in the spring 2020 semester. After seeing students’ high engagement following the communication of this policy, teachers reflected on the fact that students continued to participate and show a desire to learn despite this policy. For example, one teacher described:

It seems like we are always quick to say students do not want to learn, or that they do it for a grade. We assume they are unmotivated. But my students have shown me that despite feeling unmotivated at times, and despite the fact that grades do not really matter, they want to learn. They want to watch videos about chemistry and understand what an endothermic or exothermic reaction is. And perhaps they do not want to learn chemistry, which then would lead me to think they are doing their assignments out of loyalty to me – which would just show me the depth of respect they have for me. That just means that I have no regrets about the ways in which I engaged with students to build relationships.

These reflections demonstrated an acknowledgment that students are intrinsically motivated to learn, either out of genuine interest in the content or out of respect for the teacher rather than due to grades; for some teachers, this was a new revelation.

In contrast, other teachers viewed student motivation largely from a deficit perspective. One teacher wrote, “Motivation and accountability were oftentimes lacking in those scholars who chose not to complete assignments.” This sentiment was also demonstrated in another teacher’s statement that, “Creating lessons online was exciting until students put in minimal effort on activities. While instructions explicitly explained to students to write in full sentences or watch certain videos, it was not done.” Notably, most of these teachers taught in Title I schools with high populations of minorities and English Learners. Given the disproportionate impact of COVID-19 on minority and low-income families and communities, it is also likely that the students faced more significant hardships related to the pandemic than White and affluent students did. Rather than considering the broader context and wide range of challenges and inequities associated with COVID-19, some teachers blamed students for their lack of engagement. Notably, this was not the case for all teachers. For example, one described, “In-person and online teaching have shown me how resilient my students and I can be and how important it is to be adaptable to any situation.” This asset-based view of students stands in stark contrast to the views of some other teachers.

#### Unique opportunities related to the COVID context

Some teachers reported finding a reprieve through online instruction. One wrote, “Because the difficulties I had with classroom management are significantly less of an issue in an online class, I am able to focus more on ways I can engage students and make class exciting for them.” Classroom management was a common challenge and concern for these teachers, so eliminating the complexities of in-person student interactions resulted in less stress for those teachers.

Participants reflected on their work-life balance pre- and post-COVID, with many finding more time for themselves during online instruction. One wrote, “This transition has also been incredibly beneficial for work-life balance. Since I live with my family again, I limit work to school hours, yet I have been able to get more tasks accomplished in this time.” Another teacher explained, “While this pandemic makes me worry constantly about my students and just about the financial stability that millions are going through – it also allowed me a time to breathe, to relax, and to do the simple things.”

In addition to fewer struggles with classroom management and improved work-life balance, teachers also reflected on unique opportunities for leadership they were afforded because of COVID-19. As first-year teachers with limited preparation to teach, many of the teachers struggled with imposter syndrome and feeling inadequate in comparison to more veteran teachers during the majority of the school year. However, ERT afforded them new opportunities to be recognized and even take on leadership roles based on their ability to adapt to online instruction, and in particular, their tech-savvy natures. One teacher described a role reversal and how she was recognized by both administrators and other teachers for her expertise in developing a class site for online learning. Another teacher reported becoming a go-to resource for technology support in his school. In one of the most profound opportunities to be viewed as an expert and leader, one of the participants described being asked to lead professional development for other teachers.

### Phase 2: use of technology to enact leadership practices

With technology-related leadership opportunities emerging as a unique element of teaching during COVID-19 among first-year teachers, we explored these opportunities in more detail through in-depth interviews with Harris and Morgan (pseudonyms), two teachers who indicated they had unexpected leadership opportunities in their written reflections.

Harris had a bachelor’s degree in biochemistry and aspired to attend medical school following his two-year TFA commitment. He was a seventh-grade science teacher at a Title I middle school in a large urban school district in the South-Central United States. His student population was approximately 87% Latinx and 13% Black. From March 2020 through the end of the academic year, Harris taught entirely online. He also started the 2020–2021 school year with online instruction, shifting to a hybrid mode in October of 2020. In this hybrid mode of instruction, Harris simultaneously taught students attending in-person and online. All students, including those attending in person, logged into the class Google Meet session. This hybrid instructional approach continued through the end of the 2020–2021 academic year.

Morgan came from a family of teachers and had long aspired to base her career on working with children. She had a bachelor’s degree in social work that she credited with making empathy an integral part of her thought process. Morgan taught at a Title I middle school in a large urban school district in the South-Central United States. She spent 1 year as a seventh-grade science teacher at this school, then shifted to teaching eighth-grade science for the next 2 years. Morgan’s student population was approximately 66% Latinx, many of whom were English Learners, and 34% Black. Morgan noted that the zip code of her school is the second highest contributor to the prison system in the state, so many students had some experience or connection to the prison system. Like Harris, Morgan taught entirely online from March 2020 through the end of that academic year. She also started the 2020–2021 school year with a month of online instruction before shifting to a hybrid format, with students simultaneously attending in-person and online, for the remainder of the school year.

#### Domain 1: establishing and conveying the vision

As schooling shifted to ERT approaches, there was a need for a clear vision, with goals and expectations related to online instruction and the use of technology for both teachers and students. Harris reflected on how his school’s vision for instruction shifted due to COVID-19 and how that vision was communicated with teachers and students. He said, “The biggest challenge has definitely been communication in all regards.” There were few goals or performance expectations related to the use of technology, and the vision and expectations that did exist shifted over time.

Similarly, Morgan found the lack of a clear vision to be extremely frustrating. She said:

This transition was a nightmare. I wish my school had taken an extra week to figure out how to do online learning before we presented it to kids. There were too many moments the first couple weeks where expectations were constantly changing, and students were forced to adapt. I was frustrated, I hated constantly changing things up on my students, I felt like my integrity in the eyes of students was being shaken. This could’ve been fixed by figuring things out before bringing the kids in, but that wasn’t the case.

Morgan described the vision at her school continuing to shift over time, with various requirements and restrictions emerging at different times. Because administrators wanted to avoid overwhelming students and their families, they restricted teacher-student communication following an initial push to contact families to make sure students had internet access. Morgan struggled greatly with this:

It’s also really difficult with students considering my school has limited how I can communicate with my students. It’s been really upsetting to lose relationships with students because online learning has made communicating with students difficult. I miss them so much. The kids made me want to go to work, they kept me there, and to lose time with them is heartbreaking. It’s also difficult to make sure they’re understanding the content.

Reflecting on her inability to connect with students, Morgan also recalled, “We weren’t even required to do live sessions that first year. We just had kids do assignments online if they wanted some source of normalcy.” There was greater structure in synchronous online teaching in the 2020–2021 school year, with a clear schedule of when students would log in for class each day. However, inconsistent messaging remained, and in some cases became more problematic as administrators directed teachers on how to focus their attention. Morgan described:

At times, admin would come in and tell us we needed to focus on our in-person students, even though more than half of students were online … I like to think I’m pretty emotionally intelligent, so I tried to put myself in my principal’s shoes and understand where they were coming from, and even in trying to do that, I knew there were still going to be issues.

With an unclear and ever-shifting vision for instruction during the pandemic, Morgan perceived teachers at her school as not fully invested in meeting the expectations laid out for them. Morgan explained:

At my school, a lot of teachers feel extremely jaded and unseen and unheard. And since they feel that way, they don’t follow expectations that the school laid out that could make them more successful. It makes it really difficult to care about data when you don’t feel seen or heard by your school.

Despite teachers and students growing accustomed to technology teaching resources, Morgan explained that her administration decided electronics would not be allowed in the classroom at the start of the 2021–2022 school year.

#### Domain 2: facilitating a high-quality learning experience for students

Both Morgan and Harris sought ways to utilize technology to promote student engagement in ERT and to maintain an organized and safe online learning environment. Morgan reported spending considerable time and effort thinking about effective online instruction, and she even completed an action research project inquiring into her effectiveness in the hybrid setting, with students participating in class activities both in-person and online simultaneously. As part of this project, she reviewed the literature on online teaching effectiveness. She said:

Everything that I read said yes, you can be extremely effective as an online teacher, just as effective as you can be as an in-person teacher. But there was literally nothing out there that I could find that talks about science in a hybrid setting where you have both in-person and online students. So, I don’t think being online hindered my ability to teach; I think being hybrid hindered it.

Although she saw the potential for quality online instruction, Morgan remained skeptical about the hybrid approach. She described using a number of technology tools in her efforts to create an engaging learning experience for students, including Pear Deck, Zoom, Edpuzzle, Kahoot, Quizizz, and PhET simulations. She liked Pear Deck so much that she actually envisioned using it every day even with students attending school in person. She explained, “Ideally, I would use Pear Deck every day, even with students all attending in person. I loved it. I was able to check student work really efficiently as they were working.” She also appreciated the communication affordances of Zoom and saw her students who had been less vocal in person engage to a greater extent because of the private chat feature. Morgan described her use of Zoom:

I wish that I could have the hybrid style [teaching both in-person and via Zoom], but with every kid actually in the classroom. I loved the ability to have private conversations over Zoom, like if a kid was acting outrageous in the classroom, I messaged them on Zoom instead of calling attention to their behavior in front of everyone … but I desperately wish they were all in person for things like being able to look at their paper and point to issues, or doing fun hands-on science activities.

There was some overlap in the technology tools that Harris described using. He reported most consistently using Nearpod and Google Classroom, but he also used Edpuzzle, Quizizz, Google Forms, Blooket, and Quizlet for instruction and assessment purposes. After having taught in this format for over a year, Harris reflected, “I have a better idea now of what online tools students themselves enjoy using, making them more invested in the learning.” Similarly, Morgan shared, “I have learned so much about technology and various websites to utilize, and I know they could be transformational if I’m in the classroom while they are using them. These online resources will also be helpful to differentiate.”

Both Morgan and Harris described a greater emphasis on student engagement and enjoyment than prior to the pandemic, due to a variety of factors. First, they found classroom management demands were reduced. Harris described:

As a younger, more tech-savvy teacher, online learning has been a great opportunity for me. Because the difficulties I had with classroom management are significantly less of an issue in an online class, I am able to focus more on ways I can engage students and make class exciting for them.

Morgan similarly discussed relief from classroom management pressures with online instruction. She said, “Classroom management was definitely easier online and hybrid. That doesn’t mean it was always easy or that it was perfect, but it was easier.”

Second, they described the removal of some of the typical teacher accountability measures also contributing to an increased focus on student engagement. Harris explained:

Both last year and this year, we didn’t have standardized testing. So, I was like, I can really do whatever I want … I definitely think we test our kids way too much. I get the desire for data, but I think the people who administer those tests are not fully honest with themselves about the costs of the data in terms of the emotional and mental toll on students. The AP [assistant principal] who ran our PLC [professional learning community] wanted to have a data-driven celebration at the beginning of each PLC, and I was like, “That’s ridiculous. I think it’s ridiculous that you only want my celebrations to be driven by data. And I think it takes out the humanity of students a lot.”

With fewer standardized test pressures, Harris felt he had more flexibility to teach skills that extended beyond science content standards. He recalled:

I remember Jeanna mentioned during our class that she was working with the state committee to revamp their science standards, and she said one of the baseline principles was that nothing we list as a standard can be easy to Google, and I looked at my standards, and I thought, “Every single one of these are facts that can be Googled, so I need to teach my kids a different skill set.” Teaching online has also lent me the flexibility to teach students more digital literacy skills such as typing and creating a presentation. I am using this time to try and give them more explorative opportunities, which I think is at the core of STEM learning.

Harris also found technology to improve the learning experience for students because it supported a more organized approach. He shared:

Something I struggled with in my first year of teaching was organization, so having everything on the computer [during ERT] was super easy. I would just send them the link, I knew where everything was, it was a lot easier to check if they actually did something … doing everything digitally is natural documentation because they can’t delete their work like they might lose a paper version.

While Harris appreciated this level of organization for his own purposes, Morgan also saw benefits to students when she used a clear and consistent structure for her Google Classroom site, which also included pictures and visuals to make it more similar to her in-person classroom. She described:

The biggest scare I saw in quarantine was the fear of not knowing and the fear of not being able to access knowledge, information, food, whatever. My hope was to give as much information as possible and as much normalcy as possible, which wasn’t forcing kids to do work but was more like helping them remember our classroom setting and voicing that I was available, giving them a million different ways to communicate with me … and paying attention to social and emotional learning.

Although both Harris and Morgan found technology tools to be central to their instruction and useful in facilitating student interaction, they did report struggling with keeping students engaged, particularly for those attending class online rather than in person. Harris said:

At the beginning of the 2020–2021 school year, I probably had 40 to 50% of students in person. And by as early as November, I maybe had like 20 to 25%. I think both teachers and students were pretty exhausted, so I wouldn’t say our level of engagement online was great. I would say engagement was high among students who came to class in person. They just had a lot fewer distractions with so few other students there.

Similarly, Morgan described:

There was a very stark difference, where those kids who would come to school in person, even if they hated being there, you can at least talk to them and have some sort of common ground with them. But if these students attended online, it was like they just dropped off the face of the planet for us. Some of those kids that we completely lost all contact with, they didn’t come back to school the next year.

Thus, Morgan and Harris described utilizing technology tools to make ERT as engaging as possible for students, but they faced limitations in achieving ongoing engagement.

#### Domain 3: building professional capacity

Technology-focused professional learning opportunities were much needed as teachers shifted to online and hybrid instruction, but participants reported that these opportunities were rarely provided by school administrators. In addition, support for and recognition of exemplary technology use was inconsistent. Harris described himself as relatively comfortable with technology and troubleshooting problems based on his knowledge of what should be possible with technology. He explained, “My know-how with technology helped me, and it wasn’t something that I had learned, it was just because I’m more familiar with how the systems integrated with each other.” Based on this technological know-how, Harris reported identifying solutions to problems he and other teachers faced. For example, he used a plug-in for Google Chrome that allowed him to automatically transfer grades from Google Classroom to his school’s learning management system. He used a Google Form to collect student and family information, which could then be automatically uploaded into TalkingPoints, an app he used to communicate with families. He also discovered a plug-in that could be used to insert prepared slides into students’ individual Google Slides files that served as their interactive science notebooks, providing them with additional structure and prompts within their notebooks.

Knowing that his colleagues faced similar challenges, as well as more basic technology issues, Harris described starting a YouTube channel, where he posted technology tutorials for other teachers. He said:

When the pandemic happened, I actually made YouTube videos that I sent to other teachers at my school, on how to set up different online tools, import grades, use OneNote, which was on all of our computers. I set up Microsoft Teams for our school because before that the only means of communication was just sending emails on our full school listserv, which actually drove me insane … My administration never asked me. I just had all this time, and I saw my colleagues not understanding things, so I would just record a video to show them.

Notably, Harris described taking it upon himself to adopt the leader practice of building professional capacity among his colleagues. He saw this type of initiative as largely absent at the school level, but teaching during COVID-19 left Harris with hopes for the future related to technology integration and professional development. He explained:

I think school and district administration now sees the necessity of having digital skills and will prioritize this as part of standards that students meet, along with content knowledge. This may result in teachers also taking required professional development courses on different digital literacy skills, such as typing, Google Drive and Google Classroom, and different resources to integrate into their normal everyday class.

Like Harris, Morgan described emerging as a technology leader in her school, with her school administrators sharing her Google Classroom as an exemplar for other teachers. At the conclusion of her first year of teaching, Morgan described:

My biggest success during this time has been with my Google Classroom, specifically in the eyes of my administration... Since I have put this effort in, my administration has used my Google Classroom as an exemplar in meetings. Everyone at my school has seen my classroom, and I have other teachers reaching out to ask me for help with their classrooms. After being in the classroom where no administration ever came in, where I rarely had any voice and help, I’m now in the exact opposite position and it feels really good.

As she entered her second year of teaching, Morgan reported her ongoing efforts that her school administrators saw as going above and beyond the expectations. She explained:

A lot of the praise I’ve gotten from my school has been around things that I take extra time to do. Last year I was heavily involved in the process of getting my students applying for different high schools with better college preparation … and I got praise in weekly newsletters for those types of things.

While Morgan reported being recognized for her efforts, she still struggled with feelings of inadequacy, even as she entered her third year of teaching. Morgan described:

I had major imposter syndrome when I started teaching. I didn’t feel like I belonged or like I should be there, and I guess I still struggle with a sense of inadequacy because I feel like my students deserve so much more than me, but now I recognize that regardless, I can still show up for them, and I’m going to do that. My place is a White woman in a classroom where I am the only White person in the room, and I know I’ve been molded to be racist, and I’m constantly working against that … but a lot of my imposter syndrome was not understanding how I can be a source of structure and management without my Whiteness being part of it. I feel like I’ve grown, and I’m more comfortable commanding a room in a way that’s inclusive and comfortable. I feel like I’m a stronger person.

#### Domain 4: creating a supportive organization for learning

Leader practices in this domain included acquiring and allocating technology resources to teachers and students, attending to equity in technology access, distributing technology-related leadership, maintaining high expectations for technology integration, and supporting teachers in collaboration. Although Harris and Morgan described being able to access the technology resources they wanted to use in their teaching, they saw this as largely because they adopted the leader practice of acquiring technology themselves rather than because of concerted school efforts to provide access. For example, Harris reported putting forth a lot of effort to develop instructional materials that were useful in online and hybrid teaching. He recalled, “My co-teacher and I were creating everything from scratch. We had nothing made for us, not a single test or assessment provided by the district, so we had to make everything or source everything.” As such, the expectations for technology integration were unclear. While Morgan reported having similar issues with not being provided with online and hybrid instructional materials, she did not have the benefit of working with another teacher of the same course. She described, “Year one was extremely brutal. There were a lot of things going on, and I didn’t teach those subjects with anyone.”

In addition to these organizational issues with providing teachers with online instructional resources, Morgan and Harris also identified problems with ensuring equitable student access to technology. From a philosophical point of view, Morgan emphasized the importance of access, saying, “Students deserve access, they need to be more technologically advanced in this world and we can give them this opportunity.”

Harris agreed with Morgan’s sentiments and reflected on the equity issues exposed or reemphasized by COVID:

This pandemic has truly exposed the inequities in a variety of systems, especially education. One hope I have coming out of this is that the necessity of universal access to the Internet is further emphasized. Many students have struggled with learning even if they want to due to expensive internet costs, and COVID-19 has underscored that disparity.

After over a year of online and hybrid instruction, Morgan was pleased to see that some of these basic access issues had largely been overcome, saying, “We once thought it would be impossible for all our students to have access to internet and laptops, but here we are, with the majority of our students safe at home, doing their work.” However, she still struggled in thinking about the extensive inequities students face and what her position was in combating them:

It’s easy to blame myself for not being successful and not creating an equitable environment for my students, but I also have a set of systems that I’m working within, which is my assistant principal, who works under the principal, who answers to their executive directors, and whatever level. So, I struggle to fully understand how we can make our school environments more equitable because we need to talk about the big picture, which is that our system needs to be completely overhauled. And not just the education system, but how we view the entire democracy … but I do think that there is a micro system at play that can be fixed to make things better at the classroom level.

Simultaneous to these concerns about equitable student access to technology, Harris saw new leadership responsibilities being distributed to him because of the newfound importance of technology. He reflected:

My familiarity with technology has allowed me to connect more with other teachers and almost reverse my role in which now I am the expert and they are the novice teacher. Using virtual tools is much more in my comfort zone, and it has been great for me to be able to teach it to other teachers on my campus … Compared to my colleagues, I would say I was much more effective teaching online than they were … It was odd for me because I suddenly became kind of like a master teacher in certain areas because I was just a lot more comfortable with the medium. The veteran teachers who had been teaching for 20 or 30 years, they were much less comfortable with technology, so I was like, “Okay, stop by my room, and I will show you whatever you need.”

Harris found these leadership opportunities and recognition to be very beneficial, particularly as an early-career teacher with few other leadership opportunities. He shared:

Title-wise or in any technical way, nothing changed in terms of my leadership. But teachers definitely came to me a lot more, and being the resident science teacher gave me some degree of credibility, even for talking about COVID vaccines.

Thus, although there was no formal recognition of Harris, he appreciated the opportunity to be seen as a leader and resource among his colleagues.

Although Morgan did not describe being sought out by her colleagues for support in the same way that Harris did, she also saw herself as a more effective online and hybrid teacher than her peers, largely because of her commitment to her students. She explained:

Compared to my colleagues, I think I was more successful. I can’t say that for everyone at my school, but generally I was never a teacher who left my computer … I was definitely very invested in my online kids, and whenever I would have conversations with my coworkers, it seemed like they didn’t care. They would just completely walk away, and they had no clue how their kids online were doing. I was committed to my students, and I was not about to be like, “Hey, come to my class so I can count you present, but sink or swim!” I couldn’t do that.

Despite being early-career teachers, both Harris and Morgan described gaining new perspectives about their strengths and assets as teachers because of the pandemic and their observations of their colleagues.

#### Domain 5: connecting with external partners

Harris and Morgan reported adopting new leader practices in the face of the pandemic to utilize technology to build relationships with families and to engage them in collaboration to improve student learning outcomes. In fact, some unexpected benefits in terms of fostering communication emerged. Though her school limited how she was able to connect with students, Morgan shared her experiences reaching out to families to navigate online instruction:

My communication with families increased because of the pandemic, especially when quarantine just began. We were forced into a situation where no one knew what was going on. A lot of my kids didn’t have Wi-Fi, so my school asked us to call everyone in our first hour, and that allowed for a deeper conversation and relationships … It led to deeper and more meaningful relationships with students and their families than I could have imagined.

Harris also saw deeper relationships forming with students, but he specifically relied on technology to foster these relationships. He described, “It has also given us clear ideas of how we can leverage technology to enhance learning and communication in our communities not just during online learning but when classroom learning resumes again as well.” In particular, Harris utilized the TalkingPoints app with his students and their families. He explained:

I have a lot of Spanish-speaking students and Spanish-speaking parents, and one of the things I did this year [2020–2021] that I didn’t do last year and I wish I had is use TalkingPoints. It was a way for me to send messages to parents, and it had an automatic translation feature. Ironically, I had way more parent engagement than I did last year, and I found that the texting [using TalkingPoints] was way better than phone calls because a lot of parents work, so trying to get them to pick up the phone during the day was hard. Throughout the year, I would estimate I had probably at least 80% of parents engage with my messages at some point.

Harris saw great value in using this app to build relationships with families, opening the door to collaboration with them to ensure the success of their student. He recalled:

Advice I remember getting before I started teaching was not to make your first interaction with the parent some discipline issue that you had. And so, my first couple of messages were just announcements. If I messaged them later with concerns about not getting any response from their student, a lot of parents told me, “You’re the only person who I’ve gotten in touch with at the school.” So that was really big for me.

Harris took great pride in being accessible to parents, and he described prioritizing responsiveness to their messages, even responding late at night to show he was available and solidify these relationships.

## Discussion

The phenomenon of transitioning to ERT during COVID-19 represented a significant shift in relation to technology use and leadership opportunities for early-career teachers. During their first 6–7 months of teaching, these individuals were surrounded by teachers with more experience and expertise, with opportunities to discuss teaching practice through both formal structures (e.g., Professional Learning Communities, teaching observations) as well as informal conversations. However, starting in mid-March of 2020, typical teaching and leadership norms were no longer applicable. Practices continued to shift throughout the 2020–2021 academic year, with in-person, fully online, and hybrid approaches used at various times. Teachers needed to be nimble and responsive to this new educational landscape.

The teachers in this study perceived themselves as better situated to take on the challenges associated with ERT than many of their veteran teacher colleagues, which led to new leadership opportunities within their schools and districts. Although prior studies have produced conflicting findings related to the extent to which digital natives are better equipped than more veteran teachers in integrating technology into their instruction (e.g., Goos, 2005; Lei, 2009; Russell et al., 2003; Tondeur et al., 2017), specialized knowledge in technology integration can present unique teacher leadership opportunities, even among early-career teachers (Gao et al., 2010; Gao et al., 2011). Indeed, in this study, TFA corps members had opportunities to lead and be recognized for that leadership within their schools and districts. With high rates of attrition among TFA teachers (Heilig & Jez, 2010) and previous research noting that leadership opportunities can support teacher retention (e.g., York-Barr & Duke, 2004), these opportunities may be particularly valuable for TFA corps members. In addition, peer learning approaches can also benefit teachers with less technological expertise (Dexter & Richardson, 2020), capitalizing on distributed leadership skills (Lieberman & Friedrich, 2010).

While the transition to ERT presented challenges, many of the participants reported feeling like teaching was in some ways easier or more manageable in this new format. In fact, some even reported experiencing positive impacts on their stress levels and work-life balance. Notably, research on teacher stress during the pandemic has produced mixed findings. Similar to this study, Herman et al. (2021) found that teachers reported experiencing less work-related stress after the onset of COVID-19 compared to their pre-COVID stress levels; in particular, confidence in classroom management was associated with greater teacher well-being. In exploring the return to school in fall of 2020, Pressley et al. (2021) found that teachers conducting virtual instruction experienced the greatest anxiety and stress. However, it is also important to consider individual characteristics associated with stress during the pandemic. Women, and mothers in particular, were more likely to face additional labor and stress at home due to COVID-19 (Hjálmsdóttir & Bjarnadóttir, 2021; Santamaría et al., 2021). As early-career teachers who had recently completed their undergraduate degrees, most of the participants in this study were unmarried and had no children; thus, their home circumstances may have been different from other teachers, resulting in their improved work-life balance.

In addition to finding greater work-life balance, suddenly, participants’ expertise and comfort with utilizing technology for instruction became a valuable asset for teaching online. They found that challenges with classroom management were no longer as pressing or limiting in their work. The skills needed to teach effectively shifted away from classroom management to instead finding new ways to engage students in an online space and experimenting with effective platforms for learning. This created opportunities for early-career science teachers to develop as emerging leaders in their schools and fostered their self-efficacy and confidence. Harris and Morgan, as well as other participants in this study, reported feeling validated by the recognition they received during this time. This is aligned with the notion of providing teachers with authentic leadership opportunities to reinvigorate their teaching practice (York-Barr & Duke, 2004) and support a more positive sense of teaching self-efficacy (Day & Gu, 2007).

Several of the practices these teachers reported engaging in while teaching during the pandemic aligned with the operationalized descriptions of technology-driven leader practices outlined in Table 1 (adapted from Dexter et al., 2016; Hitt & Tucker, 2016). For the first domain, *establishing and conveying the vision*, teachers in this study described modeling specific uses of technology and providing their colleagues with a vision for technology integration, often in the absence of a strong and clear vision for online teaching at their school. Harris’s willingness to create a YouTube channel to provide other teachers with skills to integrate technology demonstrated the need for that vision within his school. The second domain, *facilitating a high-quality learning experience for students*, was evident in the work of the teachers in this study, as they reported utilizing technology to promote engagement and check in on students personally. While engagement waned in ERT compared to face-to-face instruction for many teachers during the pandemic, the teachers in this study both believed they had superior student engagement compared to their colleagues, which they attributed to their technology integration. The third domain, *building professional capacity,* was evident in instances when participants described their school leadership recognizing their exemplary uses of technology and fostering the sharing of this expertise with their colleagues. The fourth domain, *creating a supportive organization for learning,* was described when the teachers emerged without prompting to help their colleagues navigate online teaching practices and streamline approaches for students to have some level of consistency across their different teachers. The fifth domain, *connecting with external partners,* was evident in the teachers’ descriptions of utilizing technology to connect with children and families in more consistent and effective ways, such as through apps that translated messages to languages spoken in students’ homes. The circumstances of the pandemic required that teachers be in direct contact with parents to set up online learning and keep track of students during this transition period to online instruction. In addition, participants found that teaching while students were at home invited teachers to get to know their students in ways that were often more personal and connected than they were able to do during face-to-face instruction.

### Limitations

While this study provides important information about early-career teachers’ experiences with ERT, several limitations of the work must be considered. First, the participants in this study followed an alternative route to teaching by participating in TFA. This experience is distinct from the path many individuals take in becoming teachers. Second, the findings in this study are based on participants’ self-reported experiences. It is possible that fellow teachers and their school leaders may have had different perceptions of the participants’ instructional practices and leadership. Finally, because these teachers’ experiences may not be representative of all teachers in all contexts, caution should be taken in generalizing specific findings to broad populations of teachers. Probabilistic generalization, which is based on statistical sampling requirements, is not possible given the design of this study. However, theoretical generalization, which aims to refine existing theory (Eisenhart, 2009), may be possible. This approach to generalization seeks to accumulate evidence gradually and with constant comparative strategies (Davies, 1999). This study expands on the literature on technology-related teacher leadership to consider the unexpected leadership opportunities early-career teachers had during the COVID-19 pandemic. We invite additional studies that further explore our findings across contexts to continue this process of theoretical refinement.

## Conclusions and implications

Teacher leadership has been explored as an area for school reform for decades. As the work of teaching shifted dramatically during the COVID-19 international pandemic, new skills for teacher leadership emerged that had never been required before. The unique circumstances of the pandemic, requiring ERT, created a need for many K-12 teachers to dramatically rethink how teaching and learning occurred in their classrooms. New teachers described emerging as leaders during this period, paving the way for other teachers to find increased success teaching in online and hybrid modalities. The teachers in this study felt liberated by the removal of the constraints imposed on their teaching by the “testing culture” when the state assessment was cancelled. This emerging educational landscape afforded the teachers a new space to experiment and be creative with new methods and pedagogies that could be utilized to reach their learners. Teacher preparation programs should intentionally provide space for pre-service teachers to think creatively about instructional approaches and technology integration to support innovation in our system of schooling even after the pandemic.

The teachers in this study described emerging as leaders in relation to the characteristics of the unified model of effective leader practices (Hitt & Tucker, 2016). In many circumstances, they adopted these leader practices without prompting by their formal leadership team, demonstrating their desire to contribute to their schools in a way that reached beyond their individual classrooms. Although the ERT circumstances that resulted from the COVID-19 pandemic created numerous challenges for teachers, the early-career teachers in this study reported having novel opportunities to assume leadership roles related to instructional innovation and technology integration. Given the importance of leadership and recognition for teacher persistence, schools should strive to leverage the unique assets that early-career teachers bring to the profession even beyond the pandemic. Views of professional development should be expanded to recognize informal learning opportunities that already exist within schools. With the addition of some intentional structures, teachers can increasingly learn from one another. For example, communities of practice grounded in topics of shared interest (e.g., technology integration) could allow for new leaders to emerge and support shared learning. By distributing leadership practices in these informal ways, a greater range of teachers can help advance teaching practices while also receiving affirmations about their place in teaching.

ERT associated with the COVID-19 pandemic presented many challenges for teachers, students, and families. Although some of the teachers in this study described having unique leadership opportunities, they also worried about their instructional effectiveness in ERT. In particular, they found hybrid instructional approaches in which they taught in-person and online students simultaneously to be particularly challenging. While ERT served as the best possible option at the outset of the pandemic, providing access to educational opportunities that would otherwise be absent, this type of instruction is intended to be temporary. Policymakers should carefully consider the limitations associated with ERT and proceed with caution in relying heavily on this approach to teaching.

## Data Availability

The datasets generated and/or analyzed during the current study are not publicly available due to privacy concerns but are available in blinded format from the corresponding author on reasonable request.
